# Distillers’ grains as alternative feed resources for beef cattle: review

**DOI:** 10.5713/ab.250771

**Published:** 2025-11-14

**Authors:** Gercino Ferreira Virgínio Júnior, Kalista Eloisa Loregian, Danilo Domingues Millen

**Affiliations:** 1 School of Agricultural and Veterinary Sciences, São Paulo State University (UNESP), Jaboticabal, Brazil

**Keywords:** Co-products, Ethanol By-products, Feedlot Nutrition, Nutrient Excretion, Rumen Metabolism

## Abstract

Distillers’ grains (DG), including wet, dried, and dried with solubles, are valuable co-products for beef cattle feeding due to their high digestible energy, rumen-undegradable protein, and functional fiber content. This review integrates current knowledge of the nutritional, productive, environmental, and economic implications of DG use in feedlot systems across temperate and tropical regions. At moderate inclusion levels (15%–30% of dietary dry matter), DG consistently enhances feed efficiency and reduces feeding costs without compromising growth performance or carcass traits. Their low starch concentration contributes to more stable ruminal fermentation and a lower risk of subacute acidosis compared with high-grain diets. Nevertheless, excessive ether extract and sulfur concentrations may depress fiber digestibility and increase the incidence of metabolic disorders such as polioencephalomalacia when diets are improperly formulated. Environmentally, DG improves resource efficiency by recycling ethanol co-products and reducing reliance on conventional feed grains; however, their high nitrogen and phosphorus contents can elevate nutrient excretion and potential environmental load if not properly managed. Economically, DG enhances profitability for feedlots located near ethanol plants, though market volatility and transportation costs remain key constraints. Recent advances in co-product processing and fractionation have mitigated several nutritional limitations, broadening the applicability of DG in precision feeding programs. Future progress will rely on refining nutrient characterization, optimizing phase-specific inclusion, and integrating DG within sustainable beef production frameworks. When strategically incorporated, DG serve as efficient, cost-effective, and environmentally responsible ingredients for modern beef production systems.

## INTRODUCTION

The pursuit of sustainable and cost-effective beef production has intensified the search for alternative feed resources that reduce reliance on corn and soybean meal. Among these, distillers’ grains (DG), co-products of corn-based ethanol industries, have emerged as strategically relevant ingredients in feedlot systems across the Americas, Asia, and Europe. Their adoption reflects not only their high nutritional value, rich in protein, digestible fiber, residual fat, and phosphorus, but also their role in enhancing the circularity of agro-industrial systems [1].

The expansion of dry-milling ethanol plants, particularly in the United States and Brazil, has markedly increased DG supply [2]. While only the starch fraction of corn is fermented into ethanol, the remaining fractions, fiber, protein, lipids, and minerals, concentrate into nutrient-dense co-products such as wet distillers’ grains (WDG), dried distillers’ grains (DDG), distillers’ grains with solubles (DDGS), and condensed distillers’ solubles (CDS) [3]. However, this expansion has also revealed critical challenges. Compositional variability across plants and regions, high sulfur and fat concentrations, and processing-induced quality losses (e.g., heat damage during drying) may constrain their safe inclusion [4,5]. Furthermore, excessive phosphorus excretion and inconsistent effects on carcass fatness and marbling remain contentious issues in the literature.

In Brazil, the rapid growth of corn ethanol has led to DG becoming increasingly available for zebu-influenced feedlots, with inclusion levels of up to 30% WDG showing promising results for animal performance and carcass traits [6,7]. et, differences in infrastructure, climate, and feedlot management compared with temperate regions require adapted strategies to ensure efficient and safe utilization [1].

This review critically evaluates the role of DG in beef cattle nutrition, integrating evidence on classification, composition, animal performance, carcass quality, rumen fermentation, environmental implications, and economic feasibility. Beyond summarizing published data, the review highlights knowledge gaps, conceptual frameworks for DG evaluation, and future directions that align their use with the broader agenda of sustainable intensification.

In contrast to previous reviews that have primarily focused on either compositional aspects of DG or their use in monogastric nutrition and industrial applications, the present review provides a comprehensive, system-level synthesis specifically centered on beef cattle production. It integrates evidence from temperate and tropical systems, combining nutritional, productive, environmental, and economic dimensions. Furthermore, it incorporates recent advances in co-product fractionation technologies, phase-specific nutrient modeling, and sustainability frameworks that link the ethanol and beef industries. This holistic perspective establishes the distinctiveness of the current review within the broader literature on DG.

## CLASSIFICATION AND PRODUCTION OF DISTILLERS’ GRAINS

**Figure 1:**
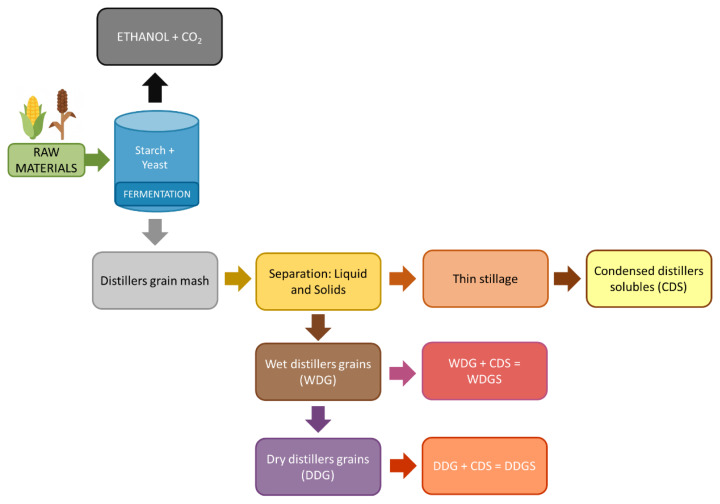
Schematic representation of the production of distillers grains as co-products of the ethanol industry.

The DG are obtained during the dry-grind ethanol production process, in which corn is ground, mixed with water and enzymes, and fermented by yeast to convert starch into ethanol and CO_2_ (Figure 1). Following fermentation, the remaining whole stillage is centrifuged to separate the liquid fraction, known as thin stillage, from the solid fraction, termed WDG. The thin stillage may be condensed into CDS, which can be reincorporated into WDG to produce wet distillers grains with solubles (WDGS). Subsequent drying of WDGS yields DDGS. The combination of fermentation, separation, and drying steps explains the nutrient concentration and variability among different DG products [8].

Although this classification is well established, the increasing diversification of ethanol plants has introduced greater nutritional variability among DG sources, with marked differences in protein solubility, lipid content, and phosphorus concentration. Such variability is not merely a descriptive issue: it has direct implications for diet formulation, animal performance, environmental nutrient management, and even market valuation of co-products. Thus, understanding DG classification goes beyond process terminology and is central to addressing their opportunities and limitations in beef cattle systems.

### Types of distillers’ grains and co-products

DG can be broadly classified according to moisture content, solubles addition, and processing intensity, factors that directly influence nutrient density, storage stability, transport feasibility, and ultimately their economic value in cattle diets (Table 1). The choice between wet or dried co-products is largely determined by the distance to ethanol plants, feedlot infrastructure, and the nutritional objectives of the production system.

### Ethanol production pathways: dry vs. wet milling

Globally, the dry-grind process is the predominant technology, accounting for more than 90% of ethanol production in the United States and Brazil [9,10]. This pathway concentrates the non-starch fractions of corn into co-products such as WDG, CDS, and DDGS, which are the focus of ruminant feeding strategies. In contrast, wet milling is a capital-intensive system that separates the corn kernel into starch, germ, gluten, and fiber, yielding co-products like corn gluten meal and corn gluten feed [11]. While nutritionally relevant, these co-products are more diversified and often integrated into other industrial chains, making them less representative of beef cattle rations compared with DG from dry milling.

From a nutritional perspective, the predominance of the dry-grind system implies that most available DG share common strengths and challenges: high protein and digestible fiber concentrations, but also considerable variability in sulfur, fat, and phosphorus contents. These process-driven differences are not trivial; they directly affect diet formulation, animal performance, and environmental nutrient excretion. Therefore, while classification provides a structural framework, the critical issue lies in managing the variability created by processing technologies, a gap that still limits the predictability of DG feeding value.

### Technological innovations and fractionated products

Recent processing innovations have led to the development of fractionated DG products with more consistent nutrient profiles and targeted applications. Examples includes:

*High-protein dried distillers’ grains (HiPro DDG):* Generated through fiber and fat separation, with crude protein (CP) levels exceeding 40% and greater rumen undegradable protein (RUP), making it attractive for finishing diets [12,13].

*Low or reduced-oil dried distillers’ grains with solubles (Lo or Ro-DDGS):* Produced after corn oil extraction, with ether extract levels reduced to 4%–6%. While this improves product uniformity and oil marketability, it also decreases dietary energy density, requiring careful reformulation [14].

*Corn fermented protein (CFP):* A refined co-product derived from enzymatic hydrolysis, with improved digestibility and amino acid balance. Although promising, its role in ruminant feeding remains underexplored, with most evaluations focused on monogastric nutrition [15].

*Fiber plus solubles (WFS/DFS):* Generated by partial removal of fiber before fermentation, yielding co-products richer in protein but with distinct nutrient profiles compared to conventional DDGS [16]. Evidence on their feeding value for cattle, however, is still scarce and inconsistent.

These emerging products represent an opportunity to fine-tune diets for protein efficiency and reduce nutrient excretion, thus contributing to environmental sustainability. However, most available studies are proprietary or preliminary, and the lack of peer-reviewed, large-scale trials hampers their critical evaluation. Consequently, while fractionated co-products expand the nutritional toolbox, their real contribution to sustainable beef production depends on robust, independent research that clarifies variability, feeding value, and economic competitiveness relative to conventional DG.

### Global production and trade

The expansion of DG is intrinsically linked to the growth of the bioethanol industry. In 2024, the United States exported over 12 million metric tons of DDGS, consolidating its position as the world’s largest producer and exporter, with Mexico, Vietnam, Turkey, and South Korea as major destinations [17]. Brazil has also rapidly scaled up its corn ethanol sector, particularly in the Center-West, where integrated ethanol–feedlot models have enhanced both the availability of DG and the sustainability of regional beef production [18].

Beyond these two leaders, wheat- and barley-based DDG contribute significantly to livestock feeding in the European Union, while China remains a key importer despite ongoing policy and biosafety restrictions [19,20]. Canada and Mexico also maintain active ethanol sectors, with Mexico consistently ranking among the top buyers of U.S. DDGS [17]. Together, these dynamics illustrate the increasing globalization of DG markets, with trade flows increasingly shaping availability and competitiveness in beef production systems.

However, the internationalization of DG supply also raises critical challenges. Variability in nutrient composition across regions, risks of nutrient degradation during long-distance transport, and regulatory restrictions related to biosafety and environmental standards complicate their consistent use in cattle diets. Furthermore, reliance on exports makes local industries vulnerable to price volatility and trade disputes. Addressing these issues requires robust quality assurance protocols, improved logistics, and region-specific feeding strategies to ensure that DG can fulfill its potential as a sustainable feed ingredient worldwide.

## NUTRIENT COMPOSITION AND VARIABILITY OF DISTILLERS GRAINS

### Overview of nutrient composition

Because starch is largely removed during ethanol fermentation, DG are enriched in protein, fiber, fat, and minerals. This nutrient concentration explains their value as feed ingredients but also underscores a key challenge: high variability across sources and batches [21,22]. Such variability is influenced by grain type, ethanol plant practices, drying conditions, and oil extraction procedures, and it complicates accurate diet formulation.

From a practical perspective, this inconsistency is more than a descriptive feature, it represents a major barrier to maximizing DG feeding value. Overestimation of protein degradability, underestimation of sulfur content, or unaccounted reductions in energy due to oil extraction can compromise cattle performance, carcass traits, and nutrient management. Moreover, the lack of standardized reporting by ethanol plants reduces nutritionists’ ability to predict animal responses reliably.

Thus, while the overall nutrient profile of DG is well established, the critical issue lies in managing its heterogeneity. Future progress depends on improved characterization methods, routine monitoring of co-product quality, and predictive systems that integrate plant-specific processing information into feed evaluation models.

### Protein and amino acid profile

The CP concentration of DG is approximately threefold higher than that of corn, typically ranging from 25% to 35% of dry matter (DM), with HiPro-DDGS exceeding 40% CP [13]. Despite this elevated protein content, the amino acid profile closely resembles that of corn, with lysine as the most limiting essential amino acid [5]. Heat exposure during the drying process can further impair lysine digestibility through Maillard reactions with residual sugars [23], exacerbating its deficiency.

In ruminants, microbial protein synthesized in the rumen generally supplies a balanced amino acid profile; however, in high-concentrate finishing diets, microbial protein yield is strongly linked to the availability of fermentable carbohydrate [24]. When DG replaces corn, they increase the proportion of RUP, with 46%–72% of CP in DDGS escaping ruminal degradation [25]. This enhances metabolizable protein (MP) supply, but the amino acid composition of that MP often remains lysine-deficient relative to requirements for muscle accretion [26]. As a result, cattle may consume diets with CP levels well above their requirements yet still experience a functional lysine deficiency that constrains lean tissue growth [27]. This imbalance helps explain inconsistent feed efficiency responses to DDGS inclusion and has prompted research on rumen-protected lysine supplementation [28]. While supplementation appears promising, its efficacy depends on accurate estimates of lysine bioavailability and requirements.

Another challenge with DG is protein oversupply. When MP intake exceeds animal requirements, surplus amino acids undergo deamination, with carbon skeletons oxidized for energy and nitrogen primarily excreted as urea [29]. This process is energetically inefficient, as deamination incurs an ATP cost and generates heat, thereby reducing the net energy available for growth [24]. From an environmental perspective, greater urinary nitrogen excretion increases ammonia volatilization, nitrous oxide emissions, and nitrate leaching, raising sustainability concerns in feedlot systems [30]. Therefore, diets containing DG may simultaneously oversupply protein while undersupplying key amino acids, ultimately reducing nitrogen-use efficiency and intensifying environmental burden.

### Fiber fractions and carbohydrate dynamics

Neutral detergent fiber (NDF) in DG typically ranges from 33% to 44% of DM, originating mainly from pericarp and cell wall residues remaining after starch fermentation. Although DG fiber is generally more digestible than forage fiber, contributing fermentable energy and bulk to the rumen, its physical effectiveness is considerably lower because the fine particle size reduces chewing and salivary buffering compared with long forage fibe [31]. As a result, DG NDF does not sustain rumination activity to the same extent as forage-derived fiber, even when included at high dietary levels.

Residual starch in DG is usually <5% of DM, lowering the risk of starch-driven acidosis when they replace corn in finishing diets. However, the limited capacity of DG fiber to stimulate chewing implies that diets with restricted forage still require supplemental physically effective fiber to prevent subacute acidosis and maintain rumen motility.

In practice, DG should be regarded as a source of highly digestible NDF that reduces starch load, but not as a functional replacement for forage NDF in terms of rumination efficiency. This distinction is particularly critical in feedlot systems with minimal forage inclusion, where balancing fermentable energy with adequate physically effective fiber remains essential.

### Lipid content and energy value

DG traditionally contain 8%–12% fat (DM basis), although values can range from 6% to 16% [4,32]. The lipid fraction is primarily unsaturated, dominated by linoleic acid (C18:2), which can constitute ~50% of total fat [33]. This enrichment contributes substantially to the energy value of DG, with total digestible nutrients (TDN) often exceeding that of corn grain [24].

However, high concentrations of unsaturated fatty acids can adversely affect ruminal fiber digestion. These lipids inhibit fibrolytic bacteria such as *Ruminococcus flavefaciens* and *Fibrobacter succinogenes* [34,35], and require biohydrogenation by ruminal microbes, which consumes reducing equivalents otherwise available for carbohydrate fermentation [36]. In addition, lipid coatings around fiber particles can physically hinder microbial colonization, further reducing fiber degradability [37].

Technological advances have led to the production of “reduced-oil” DDGS (3%–6% fat) [14], which lowers energy density but enhances nutrient consistency and mitigates inhibitory effects of unsaturated lipids on fiber fermentation.

Thus, lipid content in DG represents both an opportunity and a limitation: it increases dietary energy supply but may compromise fiber digestion and ruminal efficiency if unsaturated fatty acid levels are excessive. Optimal inclusion requires careful balancing of energy supply, fiber digestibility, and associative effects, highlighting a need for region- and batch-specific formulation strategies.

### Mineral concentrations and safety concerns

The DG contain considerably higher mineral concentrations than corn, particularly phosphorus and sulfur, which require careful management. Phosphorus typically ranges from 0.44% to 1.58% of DM and is largely present in non-phytate, highly bioavailable forms due to fermentation [38]. While this reduces the need for inorganic phosphorus supplementation, oversupply increases fecal P excretion, contributing to eutrophication in regions with intensive livestock production [39].

Sulfur content is highly variable (0.22%–0.85% DM), influenced by the use of sulfuric acid in ethanol processing and mineral content of process water [5]. Excess dietary sulfur can lead to hydrogen sulfide accumulation in the rumen, increasing the risk of polioencephalomalacia (PEM) and impairing performance [40]. High sulfur intake may also antagonize copper and other trace mineral absorption and alter ruminal fermentation patterns. Consequently, dietary sulfur from DG is generally recommended to remain ≤0.44% DM [24].

Variability is also observed for sodium, potassium, and other trace minerals, affecting dietary cation-anion balance and potentially influencing dry matter intake (DMI) and acid-base status [41]. These characteristics illustrate that, although DG are rich in essential minerals, their variability introduces both nutritional and environmental challenges. Strategic monitoring and region- or batch-specific formulation are essential to safeguard animal health, optimize performance, and minimize environmental impact.

### Factors driving variability

The nutrient composition of DG is inherently variable, influenced by multiple, interacting factors:

*Grain type*: Corn DDGS dominate in North America, whereas sorghum- or wheat-based DDGS, more common in other regions, typically contain lower protein and fat but higher fiber [42,43].

*Fermentation efficiency*: Greater starch conversion increases CP concentration and alters the overall nutrient balance [8].

*Drying conditions*: Excessive heat promotes Maillard reactions, reducing lysine digestibility and increasing acid detergent insoluble nitrogen (ADIN), thereby impairing protein and fiber utilization [44].

*Solubles addition*: The proportion of thin stillage reincorporated during DDGS production directly affects fat and protein content [45].

*Oil extraction*: Low-oil DDGS (≤6%–9% fat) have reduced metabolizable energy (ME; ~1,975 kcal/kg) compared with high-oil DDGS (~3,137 kcal/kg) [46].

*Plant-to-plant differences*: Variations in equipment, fermentation protocols, and quality-control measures contribute to significant intra- and inter-plant variability [47].

Comparative analyses of DDG, DDGS, and WDG illustrate the practical implications of this variability (Table 2). Data from near-infrared spectroscopy (NIRS), the Brazilian CQBAL feed database [48], and NASEM [24] highlight substantial differences in nutrient profiles: CP ranges from 29.4% (WDG–CQBAL) to 36.9% (DDG–private lab), NDF from 31.5% (WDGS–NASEM) to 67.5% (DDG–CQBAL), and fat from 5.3% (DDG–CQBAL) to >10% (NASEM).

This heterogeneity demonstrates that reliance on tabular averages may compromise diet precision and animal performance. To mitigate these risks, frequent laboratory analyses and rapid evaluation methods such as NIRS are recommended for more accurate nutrient characterization, supporting precision feeding strategies that optimize protein, fiber, and energy utilization while minimizing environmental impact [8].

## ANIMAL RESPONSES TO DISTILLERS’ GRAINS INCLUSION IN FEEDLOT DIETS

### Effects on feedlot performance

Incorporation of DG into finishing diets is widespread due to their nutrient density and economic advantages. When properly balanced, DG can enhance growth performance and feed efficiency while reducing feed costs. However, responses are highly context-dependent, varying with co-product type (wet vs. dry, reduced-oil vs. conventional), inclusion level, diet composition, and cattle genotype.

DMI is one of the most sensitive responses. Moderate DG inclusion (20%–30% of diet DM) generally maintains or improves intake and performance [1]. Excessive inclusion, particularly of high-fat or high-sulfur DG, may depress intake. Nevertheless, some studies show context-dependent flexibility: Schoonmaker et al [49] reported that early-weaned steers fed up to 60% DDGS exhibited no reductions in DMI, average daily gain (ADG), or feed efficiency despite elevated CP, fat, and sulfur levels, highlighting the importance of diet composition and animal physiology.

Moisture content modulates intake and feeding behavior. Wet co-products can enhance palatability and modify meal patterns. For example, Salim et al [50] found that cattle fed DDGS or modified wet DG (MWDGS) up to 50% of diet DM achieved similar ADG and carcass outcomes, but feeding behavior differed: MWDGS-fed cattle consumed more frequent, smaller meals, whereas DDGS-fed cattle had fewer, larger meals. This underscores that moisture affects behavior without necessarily altering overall intake or growth.

Fat concentration remains critical. Conventional corn-based DDGS may contain up to 12% fat, potentially depressing intake and fiber digestibility in high-concentrate diets [51]. Reduced-oil co-products (6%–8% fat) mitigate these effects, allowing higher inclusion levels without compromising performance. Ferreira et al [52] demonstrated, under tropical conditions, improved DMI and final BW when corn and soybean meal were replaced with de-oiled WDG up to 30% of diet DM; performance plateaued or declined at 45%, illustrating the importance of careful inclusion management.

Co-product origin also contributes to variability. Sorghum DDGS supports optimal ADG and carcass deposition at 15%–20% of diet DM, but inclusions above 30% reduce feed efficiency due to fiber and fat displacing starch energy [53].

In addition to individual trial reports, a descriptive synthesis of compiled data from multiple studies further illustrates performance responses to different levels of DG inclusion. Compared with control diets (0% DG), ADG tended to increase with moderate inclusions, particularly at 10%–20% of diet DM, where gains were numerically greater than the baseline (Table 3). The DMI showed a more variable response: while some studies reported higher intakes at 15% and 40%–50% inclusion, reductions were observed at intermediate levels (e.g., 10% and 25%). Feed efficiency (G:F) did not exhibit a consistent linear pattern, but values were often similar or slightly improved at 10%–20% inclusion, whereas higher proportions (≥40%) appeared to reduce efficiency. A complete dataset with raw values and corresponding references is provided in Supplement 1.

When considering wet or modified co-products (WDG, WDGS, MWDGS), descriptive data show a broadly similar but slightly more favorable pattern compared with dry counterparts. ADG was consistently greater than the control at moderate inclusion levels (10%–30% of diet DM), with the highest numerical values observed around 15%–30% (Table 4). DMI also tended to increase at these levels, reaching peaks above 11 kg/d, while a marked reduction was noted at 25% in one dataset, indicating possible variation among studies. Feed efficiency (G:F) showed modest improvements at 25% inclusion, but values generally fluctuated within a narrow range across treatments, suggesting that performance benefits were primarily driven by higher intake and growth rather than efficiency per se. Full details of the studies included in this descriptive summary are available in Supplement 2.

In feedlot systems, the optimal inclusion of DG is not universal and must be adjusted according to co-product type, chemical composition, and management conditions. Variability in moisture, fat, and sulfur concentrations can strongly influence intake, growth performance, and feed efficiency, explaining some of the inconsistent responses observed across studies. While descriptive averages from multiple trials provide useful guidance, precision feeding approaches, such as frequent nutrient analysis and batch-specific formulation, are essential to fully capture the benefits of DG while mitigating potential risks. In practical terms, moderate inclusion levels (20%–30% of diet DM) are generally safe and support growth and feed efficiency. Wet DG forms can enhance palatability and feeding behavior, whereas reduced-oil DG allow higher inclusion rates without compromising fiber digestibility. Higher inclusion levels (>40% of diet DM) are feasible under certain conditions but require careful monitoring of nutrient content and animal responses to avoid negative effects on intake, efficiency, or carcass performance.

### Effects on grazing cattle

Supplementation with DG is widely used to enhance nutrient supply, optimize forage utilization, and increase performance in grazing systems under both temperate and tropical conditions. Overall, responses are positive, but they depend on inclusion level, co-product type, forage availability, and economic context.

*Growth performance:* Several studies demonstrate that DG supplementation increases ADG of yearlings on pasture. Wheeler et al [54] reported that DDGS at 1.6 kg/animal per day increased ADG by 0.23 kg/d relative to unsupplemented controls, with positive net returns whether applied seasonally or throughout the grazing period. Adams et al [55] showed that supplementing crossbred steers with DDG cubes partially or fully replaced nitrogen fertilization, resulting in higher body weights compared with fertilized but unsupplemented controls; however, economic analysis favored unfertilized, non-supplemented systems, illustrating the need to balance biological benefits with input costs.

*Inclusion level and nutrient efficiency:* Optimal DG inclusion is critical. In Brazil, Picanço et al [56] observed that DDG supplementation at 100–200 g/kg of supplement DM maximized intake, digestibility, and ruminal fermentation, whereas higher inclusion (≥300 g/kg) reduced fiber digestibility and increased nitrogen excretion. Dias et al [13] howed that replacing soybean meal with HiPro-DDGS (430 g CP/kg) in supplements for Nellore cattle did not affect total intake but improved protein-use efficiency by reducing urinary nitrogen losses while maintaining growth. These findings highlight that moderate supplementation can enhance nutrient efficiency without compromising forage intake or animal performance.

*Strategic supplementation:* Other studies indicate DG can support flexible feeding strategies. Hasenauer [57] observed improved ADG with modified DG in yearlings grazing smooth bromegrass. Villasanti [58] reported that blending WDGS with low-quality forage allowed reductions in forage intake (~22%) without compromising performance, suggesting potential for higher stocking rates under forage-limited conditions. While some of these reports are theses rather than peer-reviewed publications, they provide experimental evidence complementing formal studies.

*Comparison with alternative supplements:* DG consistently outperform conventional protein–energy supplements in grazing systems. Troyer et al [59] found that heifers supplemented with DDGS gained 10% more ADG at lower cost than those receiving field peas, emphasizing the economic and nutritional advantages of DG.

The DG supplementation enhances growth and protein-use efficiency in grazing cattle, particularly when applied at moderate inclusion levels (100–200 g/kg of supplement DM or 0.5%–0.8% of body weight). Higher supplementation rates can reduce fiber digestibility or forage intake, emphasizing the need for careful diet formulation and monitoring of animal responses. Economic outcomes are highly context-dependent, varying with feed and fertilizer prices, forage quality, and management strategies. In practice, moderate DG supplementation represents a strategic tool in grazing systems, providing consistent gains, improved nutrient utilization, and potentially lower costs compared with conventional protein sources. Achieving optimal results requires aligning inclusion rates with forage availability, animal requirements, and the specific economic context of the production system.

### Carcass traits and meat quality

The supplementation generally supports carcass performance without compromising dressing percentage, hot carcass weight (HCW), or yield grade. Eun et al [60] and Leupp et al [61] reported no detrimental effects on carcass weight or sensory attributes when DDGS replaced conventional grain in finishing diets.

*Subtle compositional shifts:* More detailed studies reveal nuanced changes in fat deposition and muscle characteristics. High DDGS inclusion can reduce subcutaneous fat while maintaining marbling [62] and can increase deposition of polyunsaturated fatty acids (PUFAs) in muscle [63]. This shift may influence lipid stability and shelf life, although tenderness, flavor, and overall sensory acceptability remain largely unaffected [64].

*Fatty acid profile modifications:* DG consistently alters the fatty acid composition of beef. Aldai et al [65] observed that both wheat- and corn-based DDGS increased PUFA in the longissimus muscle, with wheat-based co-products having stronger effects. These changes enhance nutritional value (higher unsaturated fat), but reduce oxidative stability, which can compromise meat color and shelf life if high-fat co-products are used extensively.

*Co-product flexibility:* Sorghum DDGS and other non-corn co-products generally produce comparable carcass outcomes [66,67]. Meta-analyses reinforce these observations: DG inclusion has no consistent impact on HCW, dressing percentage, or marbling, while predictable effects on fatty acid composition are seen with minimal consequences for sensory traits [68].

The DG can be safely incorporated into feedlot diets without compromising carcass yield or grading. The most consistent effect of DG inclusion is increased deposition of PUFAs, which represents a nutritional advantage but may reduce oxidative stability and shorten shelf life if high-fat co-products are used extensively. Variability in co-product type, inclusion level, and drying conditions can influence both fatty acid composition and subsequent meat stability, highlighting the importance of targeted diet formulation to maximize benefits while minimizing risks. In practice, moderate DG inclusion maintains carcass performance and sensory quality, whereas high-fat or high-PUFA co-products may require antioxidant strategies or adjusted storage conditions to preserve meat quality. The use of non-corn DG further provides flexibility in sourcing without compromising key carcass traits, supporting sustainable and cost-effective feedlot strategies.

### Ruminal fermentation, metabolic health, and animal welfare

The replacement of starch with DG fiber, protein, and fat significantly modifies ruminal fermentation, with implications for nutrient utilization, metabolic health, and animal welfare. Moderate DG inclusion generally maintains stable ruminal pH, mitigating the risk of acidosis commonly associated with high-starch diets. For instance, Pancini et al [53] reported that supplementing SDDGS up to 30% of diet DM did not depress ruminal pH, despite minor reductions in DM and CP degradability and shifts in nitrogen utilization.

The fat fraction of DG can impair fiber fermentation by inhibiting cellulolytic bacteria, particularly when co-products exceed 10% fat [51]. Wilson et al [69] observed that diets containing 56% WDGS increased the flow of unsaturated fatty acids to the intestine, altering ruminal lipid metabolism and reducing fiber digestibility. Such dynamics indicate that conventional energy systems may overestimate the feeding value of high-fat DG.

High dietary sulfur is another critical factor. Excess sulfur in DG can induce metabolic disturbances, including sulfur-induced polioencephalomalacia (S-PEM). Probabilistic assessments suggest that finishing cattle are particularly susceptible, with both water and feed ingredients contributing to rumen-degradable sulfur intake [70] High-S diets (0.65%–0.80% DM) are associated with reduced DMI and ADG, lighter carcasses, and lower grades, while H_2_S accumulation inversely correlates with intake and growth performance [71,72]. Sulfur source may slightly affect ruminal pH, but the potential to generate H_2_S is broadly similar across sources, including DG, CDS, sulfuric acid, sodium sulfate, and calcium sulfate [72]. Short-term variations in sulfur concentration have limited effects on fermentation, but chronic oversupply can compromise mineral status (Co, Cu, Fe, Mn, Mg, Se) and increase PEM risk [73,74]. Overall, DG are safe and effective when included at recommended levels, generally up to 30%–40% of diet DM for reduced-oil products. Moderate inclusion supports stable fermentation, efficient nutrient utilization, and animal welfare. However, excessive use of high-fat or high-sulfur co-products increases the risk of impaired digestibility, metabolic disorders, and welfare concerns, highlighting the importance of careful monitoring of co-product composition and diet formulation.

### Effects on the ruminal microbiome

Inclusion of DG in ruminant diets not only provides a nutrient-dense feed source but also exerts complex effects on the ruminal microbiome. The impact on microbial populations depends on DG type, processing (wet, dried, reduced-oil, or de-oiled), inclusion level, and the production system.

Studies indicate that DG can modulate both bacterial diversity and functional pathways. Song et al [75] reported that feeding Jiang-flavor DDGS at 25% of concentrate to Guanling yellow cattle enriched specific taxa such as Ruminococcaceae and Prevotellaceae, associated with polysaccharide degradation, and altered metabolomic pathways including carbohydrate and unsaturated fatty acid metabolism. Such changes suggest improved nutrient utilization efficiency. In contrast, reduced-oil DDGS exert minimal effects on overall ruminal bacterial community composition, despite modest increases in diversity indices, with dominant phyla (Bacteroidetes, Firmicutes, Actinobacteria) remaining stable [76]. This suggests that lipid reduction attenuates the selective pressure on rumen microbes typically induced by unsaturated fatty acids.

The lipid fraction is a key determinant of microbial shifts. Castillo-Lopez et al [77] demonstrated that higher DDGS inclusion enhanced ruminal biohydrogenation of C18:1 and C18:2, increasing duodenal stearic acid (C18:0) flow while suppressing Fibrobacteres, highlighting the inhibitory effects of unsaturated fatty acids on fibrolytic bacteria such as *Fibrobacter succinogenes* and *Ruminococcus flavefaciens* [78,79].

The WDG further illustrate the influence of co-product characteristics on microbial dynamics. Rice et al [80] observed that feeding sorghum- or corn-based WDG induced shifts in fecal bacterial structure, particularly in minor phyla like Synergistetes and Actinobacteria. Tomaz et al [81] reported that de-oiled WDG increased propionate and butyrate concentrations in Nellore bulls, concomitant with higher abundance of *Selenomonas ruminantium*, indicating stimulation of amylolytic and saccharolytic populations that favor energetically efficient fermentation and reduce hydrogen availability for methanogenesis.

Overall, the microbial response to DG is strongly influenced by product type and inclusion level. Conventional DDGS, rich in unsaturated lipids, tend to suppress fibrolytic populations and modify fatty acid metabolism. Reduced-oil or de-oiled products mitigate these effects, preserving fiber degradation while enhancing microbial diversity. WDG may preferentially stimulate saccharolytic and propionate-producing bacteria, modifying SCFA profiles and energy partitioning.

**Figure 2:**
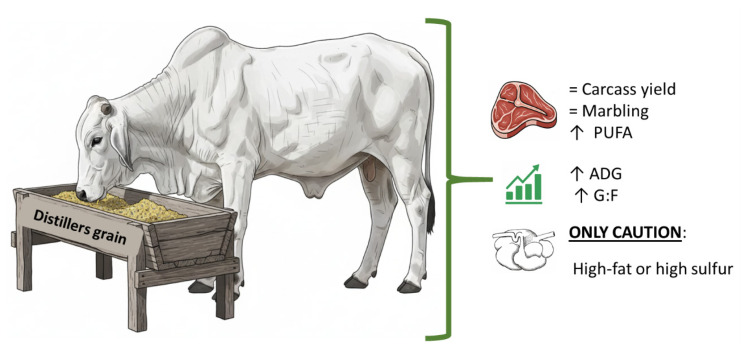
Conceptual representation of the main effects of distillers grains (DG) inclusion in beef cattle diets. Feeding DG influences rumen environment and health, which in turn affects animal performance and carcass quality. The net impact depends on inclusion level, diet composition, and animal category. PUFA, polyunsaturated fatty acid; ADG, average daily gain; G:F, gain to feed ratio.

From a practical perspective, these microbial shifts have direct implications for feed efficiency, nutrient digestibility, and methane emissions. By influencing fiber degradation, SCFA production, and ruminal hydrogen balance, DG can improve energetic efficiency and reduce the environmental footprint of cattle production. Nonetheless, responses remain variable, highlighting the need for integrated studies combining microbiome, metabolome, and performance data under commercial feeding conditions. Figure 2 summarizes the main pathways through which DG influence ruminal fermentation and productivity.

## PHASE-SPECIFIC UTILIZATION OF DISTILLERS’ GRAINS IN FEEDLOT DIETS: INSIGHTS FROM NUTRITIONAL MODELING

Nutrient requirements of beef cattle vary substantially across production phases, with protein deposition predominating during the growing stage and energy demands for fat accretion increasing as animals approach finishing [82,83]. Consequently, inclusion levels of co-products such as DDGS should be strategically adjusted to match these changing needs. Using the Beef Cattle Nutrient Requirements Model (BCNRM) [24], we simulated the effects of phase-specific DDG inclusion to illustrate potential mismatches when diets are not tailored to growth stage (Table 5).

In growing diets (300–400 kg BW), including 28% DDG aligned MP (717.6 g/d) closely with requirements (717.2 g/d), while ME (22.24 Mcal/d) met predicted demands. This balance supported an allowable gain of 1.41 kg/d, with minimal nitrogen excretion, reflecting efficient nutrient utilization. The high rumen-undegradable protein fraction of DDG (RUP; 705.45 g/d) effectively supports lean tissue deposition, while residual fat and digestible fiber contribute to energy supply.

When the same formulation was extended into the intermediate phase (400–540 kg BW), ME requirements were met (30.46 Mcal/d), but MP supply (983.7 g/d) exceeded requirements (738.4 g/d) by more than 30%. Here, allowable gain was energy-limited (1.57 kg/d), while protein could theoretically support 2.63 kg/d. The resulting imbalance illustrates inefficient nitrogen utilization, with surplus dietary protein catabolized and excreted, increasing both economic cost and environmental impact due to higher urinary N output [84]. This scenario underscores the risk of maintaining high DDG inclusion beyond the growth stage, where protein supply exceeds energy availability.

In finishing diets, reducing DDG to 15% of dietary DM and increasing corn grain to ~38% elevated dietary energy density, supporting a slightly higher ME-based gain (1.66 kg/d). Despite this adjustment, MP supply (957.2 g/d) still exceeded requirements (756.7 g/d) by ~27%, indicating persistent nitrogen oversupply. As cattle approach heavier body weights, nutrient partitioning shifts toward fat deposition [82], rendering excess MP unnecessary and energetically inefficient.

Across all scenarios, predicted methane yield remained relatively stable (~12 g/kg DM), with a slight increase in the finishing diet (12.7 g/kg DM), likely reflecting the lower dietary fat from reduced DDG inclusion. This aligns with evidence that DDG-derived lipids can depress ruminal methanogenesis [85,86].

Overall, these simulations highlight the value of DDG as a protein and energy source during the growing phase, where nutrient supply closely matches animal requirements. However, maintaining high DDG levels into finishing leads to protein-energy imbalances, inefficient nitrogen utilization, and elevated environmental risk. Adjusting DDG inclusion downward in later phases is therefore essential to optimize protein-to-energy ratios, support carcass deposition, and improve sustainability outcomes in feedlot systems.

## ENVIRONMENTAL IMPACT OF DISTILLERS’ GRAINS PRODUCTION AND USE IN ANIMAL FEEDING

The environmental implications of DG use extend beyond the ethanol production chain and directly affect nutrient cycling and greenhouse gas (GHG) emissions, and manure management. While life cycle assessment (LCA) studies indicate that DDGS can partially offset land-use change by reducing grain demand for livestock feeding, their inclusion in diets often leads to increased nutrient excretion and localized environmental pressures, particularly when fed at high levels [87,88].

### Greenhouse gas emissions

Feeding DDGS can influence GHG emissions in contrasting ways. Diets with high-fat DDGS have been associated with reductions in enteric CH_4_ yield in some cases, although effects are inconsistent across DDGS types and inclusion levels [85,86]. The proposed mechanism is that lipids, particularly unsaturated fatty acids, may suppress methanogenesis by reducing hydrogen availability for methanogenic archaea and by exerting direct toxic effects on specific ruminal microbes involved in CH_4_ formation [89]. However, the extent of this mitigation depends on the fat content and profile, which vary considerably among DDGS sources due to differences in corn processing and oil extraction practices [1]. Moreover, replacement of starch with fiber and fat in DDGS-based diets alters rumen fermentation pathways, often lowering propionate formation relative to acetate, which can counteract the potential CH_4_-suppressing effects of dietary lipids [90].

Recent evidence also indicates that reductions in CH_4_ may not be sustained at higher inclusion levels of DDGS, possibly due to adaptive shifts in the ruminal microbiome that restore methanogenesis capacity [91]. In addition, the impact on CH_4_ intensity (g CH_4_/kg of gain) rather than absolute emissions should be considered, since performance responses to DDGS can alter the denominator of this relationship [85]. Thus, while DDGS offer potential as a dietary strategy to partially mitigate enteric CH_4_ emissions, outcomes are highly context-dependent and require consideration of DDGS composition, inclusion rate, and the overall nutrient balance of the diet.

### Nutrient excretion and environmental loading

Consistent evidence indicates that DG inclusion increases intake and excretion of N, P, and sulfur (S), with implications for water and air quality [92,93].

*Phosphorus*: Manure from DG-fed cattle contains higher concentrations of total and soluble P, raising eutrophication risks if land application exceeds crop uptake. Field studies have reported increased soil P accumulation and greater crop P removal following the application of manure from cattle fed DDGS, implying that larger land areas are required for environmentally safe application [94].

*Nitrogen*: DG shift N partitioning toward urinary excretion, increasing volatile and leachable forms of N, and thereby enhancing risks of NH_3_ volatilization and nitrate leaching [92,93].

*Sulfur*: Elevated S excretion may contribute to H_2_S emissions during manure handling [40].

### Economic aspects of distillers’ grains in beef cattle production

The DG have emerged as essential co-products of ethanol production with substantial economic implications for beef cattle systems. Beyond their high protein and energy content, DG influences feed costs, animal performance, and overall profitability.

In grazing systems, supplementation with DG can partially offset lower pasture fertility or limited forage quality. Gruber et al [95] reported that steer calves grazing winter wheat pastures with DDGS supplementation had higher weight gain per hectare, extended grazing days, and improved forage utilization compared to unfertilized pastures. However, the increased feed costs associated with supplementation reduced net returns relative to moderately fertilized pastures, highlighting the trade-offs between production gains and input expenses. Similarly, Adams et al [55,96] showed that pre-finishing supplementation with DDG cubes improved initial body weight at feedlot entry, reduced feed intake and days on feed, and enhanced total system net returns. These findings suggest that strategic supplementation can improve economic efficiency, especially when producers retain ownership through finishing.

At the industry level, DG has evolved from secondary co-products into valuable feed commodities. Dennis et al [97] and Tse et al [98] emphasized that feed-use DG represents a primary revenue source for ethanol plants, with additional value obtainable through protein concentrates and other value-added byproducts. Optimizing their use in cattle diets can lower feed costs relative to conventional ingredients such as corn and soybean meal, while simultaneously improving feed efficiency and reducing reliance on synthetic fertilizers. Cross-hedging strategies using corn and soybean meal futures have also been suggested to manage price volatility in DG markets, mitigating financial risk for feedlot operators [99].

Economically optimal inclusion rates of DG in feedlot rations require consideration of multiple factors beyond feed cost, including impacts on animal performance, sulfur-related health risks, manure handling fees, and regional availability [100]. While DG can improve gross returns, enterprise budgeting studies have shown that net returns depend heavily on local pricing, logistical costs for storage and transportation, and the scale of operations [55]. Additionally, ethanol policies, subsidies, and market fluctuations influence DG availability and cost, underscoring the need for integrated economic planning.

Overall, evidence indicates that DG can enhance profitability in both grazing and feedlot systems when used strategically. The economic benefits are maximized by carefully balancing supplementation levels, feed costs, pasture management, feedlot performance, and market conditions. The integration of DG within circular agro-industrial systems further expands their value, offering opportunities for sustainability, land-use optimization, and improved resource efficiency.

## RISKS, INNOVATIONS, AND FUTURE PERSPECTIVES

The use of DG in beef cattle production offers numerous benefits, but it is accompanied by several challenges and opportunities that must be carefully managed.

*Nutritional and health considerations:* High inclusion rates of DG, particularly DDGS, can increase dietary sulfur, potentially elevating the risk of metabolic disorders, such as PEM. Additionally, variability in nutrient composition among DG sources, whether corn- or wheat-based, wet or dried, or high-protein variants, can complicate diet formulation and affect animal performance. Improper storage, especially of WDG, may also lead to microbial contamination, reducing feed quality and posing animal health risks.

*Environmental and regulatory challenges:* The elevated protein and sulfur content of DG influences manure composition, which can increase emissions of nitrogen- and sulfur-based compounds if not correctly managed. Regulatory compliance remains essential, particularly for emerging co-products, as feed safety and quality standards must be met to ensure market acceptance and confidence.

*Technological innovations:* Advances in processing and feed management are creating new opportunities for value creation. Secondary fermentation and fractionation techniques can generate protein concentrates, organic acids, and other high-value compounds, expanding the potential uses of ethanol co-products. Improvements in storage and handling, such as pelleting, extrusion, or drying, reduce spoilage and facilitate wider distribution. Integration with precision nutrition tools allows for optimization of DG inclusion rates, enhancing feed efficiency while minimizing health and environmental risks.

*Economic and market perspectives:* DG prices are influenced by corn and soybean markets, ethanol production levels, and regional availability, introducing financial variability. Risk management strategies, such as cross-hedging using commodity futures, can help mitigate this volatility. Incorporation of DG within circular agro-industrial systems not only reduces dependence on conventional feedstuffs but also enhances sustainability and operational efficiency. Furthermore, emerging co-products, such as high-protein or de-oiled DG, offer new avenues for market development; however, further research is needed to fully characterize their nutritional and economic potential.

*Future directions:* Continued investigation is needed to quantify the long-term health impacts of DG, optimize inclusion rates, and assess interactions with other feed additives. Sustainability assessments, including life-cycle analyses and evaluations of GHG emissions and land-use impacts, will support more informed decision-making. Policy frameworks and incentives can further promote the development and adoption of innovative DG products, ensuring that economic benefits are realized while minimizing risks to animal health, environmental integrity, and feedlot profitability.

## CONCLUSION

DG play a central role in modern beef cattle production, offering a versatile and nutrient-dense feed resource that enhances feed efficiency, supports rumen health, and effectively substitutes for conventional ingredients such as corn and soybean meal. Their use offers clear economic benefits by reducing feed costs and enhancing profitability, while also contributing to environmental sustainability through improved resource utilization and reduced land-use pressure. Nevertheless, careful management is required to mitigate potential risks, including nutrient imbalances and sulfur-related metabolic issues. Looking forward, continued innovation in processing technologies, precision feed formulation, and climate-smart livestock practices will be crucial to unlocking the full potential of DG and ensuring their sustainable integration across diverse production systems.

## Figures and Tables

**Table 1:** Comparative characteristics of major distillers’ grains co-products used in beef cattle nutrition

Caracteristics^1)^	WDG	MDGS	DDG	DDGS	CDS
DM (%)	30–35	45–55	88–90	88–90	20–30
CP (% DM)	28–32	28–32	26–32	27–31	18–24
RUP (% CP)	45–50	45–50	47–53	45–50	-
NDF (% DM)	34–42	32–40	33–44	30–42	-
Fat (% DM)	8–12	7–10	6–10	8–12	10–15
NEm (Mcal/kg)	2.05–2.15	2.02–2.10	1.95–2.02	1.98–2.05	1.85–1.95
NEg (Mcal/kg)	1.38–1.45	1.36–1.43	1.29–1.36	1.32–1.39	1.20–1.30
Advantages	High nutrient density, cost-effective near ethanol plants, and high palatability.	Improved storage stability over WDG; retains nutrient value; suitable for TMR.	Long shelf life; suitable for long-distance transport; consistent profile.	Higher energy density due to solubles; richer in fat, glycerol, and sulfur.	Enhances palatability, provides concentrated energy, and serves as a binder in TMR.
Limitations	Short shelf life (4–5 d); mold risk; transportation is costly over long distances.	Still perishable; higher transport costs than DDG.	Drying may trigger Maillard reactions, which increase ADIN and reduce lysine.	Greater variability; elevated sulfur may cause PEM in cattle.	Highly perishable; storage challenges; high sulfur and fat require caution.

1) Values represent typical ranges for corn-based co-products; composition varies with grain type, fermentation efficiency, and processing technology.

WDG, wet distillers grains; MDGS, modified distillers grains with solubles; DDG, dry distillers grains; DDGS, dry distillers grains with solubles; CDS, condensed distillers solubles; DM, dry matter; CP, crude protein; RUP, rumen undegradable protein; NDF, neutral detergent fiber; NEm, net energy for maintenance; NEg, net energy for gain; PEM, polioencephalomalacia; ADIN, acid detergent insoluble nitrogen.

**Table 2:** Chemical composition of different DG sources according to private laboratory (NIRS), CQBAL 4.0, and NASEM databases

Item (%)	DDG^1)^	DDG^2)^	DDGS^1)^	DDGS^2)^	DDGS^3)^	WDG^2)^	WDGS^3)^
DM	89.85	88.56	87.95	91.17	89.99	31.8	31.44
Ashes	3.51	1.93	6.06	3.43	5.32	5.77	5.13
CP	36.89	32.27	34.36	32.09	30.79	29.39	30.63
Fat	8.72	5.28	6.28	8.21	10.73	8.91	10.83
NDF	35.39	67.5	36.33	44.68	33.66	40.35	31.52
Starch	4.11	-	7.48	5.8	5.88	6.1	6.05

1) Values for DDG and DDGS were obtained from 3RLAB RIBERSOLO (Laboratório de Análises Agropecuárias S.A.; NIRS analysis).

2) Values for DDGS, DDG, and WDG are from CQBAL 4.0: Tabelas Brasileiras de Composição de Alimentos para Ruminantes (Brazilian Tables of Feed Composition for Ruminants; Valadares Filho et al [48]).

3) Values for DDGS and WDGS are from the NASEM [24].

DG, distillers’ grains; NIRS, near-infrared spectroscopy; DDG, dry distillers grains; DDGS, dry distillers grains with solubles; WDG, wet distillers grains; WDGS, wet distillers grains with solubles; DM, dry matter; CP, crude protein; NDF, neutral detergent fiber.

**Table 3:** Mean dry matter intake (DMI), average daily gain (ADG), and feed efficiency (G:F) of feedlot cattle fed diets with increasing inclusion levels (% DM) of dry (DDG, DDGS, MDDGS) distillers grains, compiled from published studies

	0	10	15	20	25	30	40	50	60
DMI (kg/d)	8.806	8.275^b^	11.600^a^	8.700^b^	8.438^b^	9.273^a^	10.400^a^	10.600^a^	9.050^a^
ADG (kg/d)	1.524	1.573^a^	1.930^a^	1.652^a^	1.388^b^	1.734^a^	1.730^a^	1.890^a^	1.740^a^
G:F	0.184	0.202^a^	0.168^b^	0.197^a^	0.177^b^	0.191^a^	0.168^b^	0.179^b^	0.193^a^

1) Values with superscript ^a^ indicate means numerically greater than the control (0% inclusion), whereas values with superscript ^b^ indicate means numerically lower than the control. No statistical analysis was performed; data represent descriptive averages across studies.

DDG, dry distillers grains; DDGS, dry distillers grains with solubles; MDDGS, modified dry distillers grains with solubles; G:F, gain to feed ratio.

**Table 4:** Mean dry matter intake (DMI), average daily gain (ADG), and feed efficiency (G:F) of feedlot cattle fed diets with increasing inclusion levels (% DM) of or wet/modified (WDG, WDGS, MWDGS) distillers’ grains, compiled from published studies

	0	10	15	20	25	30	40	45	50	60
DMI (kg/d)	9.977	9.590^b^	11.243^a^	11.400^a^	8.350^b^	11.635^a^	11.100^a^	11.375^a^	10.850^a^	10.500^a^
ADG (kg/d)	1.600	1.625^a^	1.957^a^	1.870^a^	1.300^b^	1.963^a^	1.940^a^	1.900^a^	1.860^a^	1.700^a^
G:F	0.169	0.170^a^	0.177^a^	0.164^b^	0.187^a^	0.169^b^	0.170^a^	0.169^b^	0.173^a^	0.162^b^

Values with superscript ^a^ indicate means numerically greater than the control (0% inclusion), whereas values with superscript ^b^ indicate means numerically lower than the control. No statistical analysis was performed; data represent descriptive averages across studies.

WDG, wet distillers grains; WDGS, wet distillers grains with solubles; MWDGS, modified wet distillers grains with solubles; G:F, gain to feed ratio.

**Table 5:** Composition, nutrient supply, nitrogen balance, and methane emissions in diets with varying inclusion levels of dry distillers’ grains (DDG) for growing and finishing beef cattle, simulated using the Beef Cattle Nutrient Requirements Model (BCNRM; NASEM)

Item	Growing diet	Intermediate diet^1)^	Finishing diet
Body weight	300→400 kg	400→540 kg	400→540 kg
DMI (kg/d)	8.08	11.08	10.72
Corn silage (% DM)	50	50	40
Corn grain (% DM)	15	15	37.7
Soybean hulls (% DM)	4.7	4.7	4.7
DDG (% DM)	28	28	15
Urea (% DM)	0.3	0.3	0.6
Mineral 6% P (% DM)	2	2	2
ME supply (Mcal/d)	22.24	30.46	30.89
ME required (Mcal/d)	22.24	30.46	30.89
MP supply (g/d)	717.6	983.7	957.2
MP required (g/d)	717.2	738.4	756.7
ME allowable gain (kg/d)	1.41	1.57	1.66
MP allowable gain (kg/d)	1.41	2.63	2.53
Methane yield (g/kg DM)	12.227	12.196	12.701

1) Intermediate diet represents the growing diet applied to finishing cattle.

DMI, dry matter intake; DM, dry matter; ME, metabolizable energy; MP, metabolizable protein.

## Data Availability

Upon reasonable request, the datasets of this study can be available from the corresponding author.

## SUPPLEMENTARY MATERIAL

**Supplement 1**. Raw data on dry matter intake (DMI), average daily gain (ADG), and feed efficiency (G:F) of feedlot cattle with increasing inclusion levels of dry (DDG, DDGS, MDGS) distillers grain, with corresponding references for each study.

**Supplement 2**. Raw data on dry matter intake (DMI), average daily gain (ADG), and feed efficiency (G:F) of feedlot cattle with increasing inclusion levels of wet (WDG, WDGS, MWDGS) distillers grain, with corresponding references for each study.
