# Supplementary material for: Distillers’ grains as alternative feed resources for beef cattle: review
Source: Anim Biosci. 2025 Nov 14;39(4):250771. doi: 10.5713/ab.250771 (PMC13064990; doi:10.5713/ab.250771)
Supplement: Supplementary file 1 [file ab-250771-Supplementary-1.pdf]

Supplementary 1. Raw data on dry matter intake (DMI), average daily gain (ADG), and feed efficiency (G:F) of feedlot cattle with increasing inclusion levels of dry (DDG, DDGS, MDGS) distillers grain, with corresponding references for each study

Reference	Type of DG	Level, % DM	DMI, kg	ADG, kg/d	G:F
Arias et al. (2012)	DDGS	0	8.11	1.42	0.181
		26.6	9.66	1.69	0.186
Arias et al. (2013)	DDG	0	8.23	0.83	0.163
		24.5	8.17	0.89	0.151
da Rosa e Silva et al. (2022)	DDG	0	6.08	1.338	0.22
		10	6.05	1.446	0.239
		20	6.7	1.503	0.224
		30	6.23	1.367	0.219
Salim et al. (2014)	DDGS	0	11.7	2.09	0.179
		16.7	11.6	1.93	0.168
		33.3	11.3	2.01	0.179
		50	11.4	2.1	0.185
Schoonmaker et al. (2013)	DDGS	0	9.1	1.74	0.192
		30.8	9.06	1.77	0.195
		61.7	9.05	1.74	0.193
Uwituze et al. (2010)	DDGS	0	8.02	1.58	0.195
		24	8.09	1.51	0.185
		24.3	7.83	1.46	0.185
Watson et al. (2014)	MDGS	0	10.4	1.67	0.161
		10	10.5	1.7	0.164
		20	10.7	1.8	0.169
		30	10.5	1.79	0.17
		40	10.4	1.73	0.168
		50	9.8	1.68	0.172

Abbreviations: DDG = Dry distillers grains; DDGS = Dry distillers grains with solubles; MDGS = Modified dry distillers grains with solubles;
