# Supplementary material for: Distillers’ grains as alternative feed resources for beef cattle: review
Source: Anim Biosci. 2025 Nov 14;39(4):250771. doi: 10.5713/ab.250771 (PMC13064990; doi:10.5713/ab.250771)
Supplement: Supplementary file 2 [file ab-250771-Supplementary-2.pdf]

Supplementary 2. Raw data on dry matter intake (DMI), average daily gain (ADG), and feed efficiency (G:F) of feedlot cattle with increasing inclusion levels of wet (WDG, WDGS, MWDGS) distillers grain, with corresponding references for each study

Reference	Type of DG	Level, % DM	DMI, kg	ADG, kg/d	G:F
Arias et al. (2012)	MWDGS	0	8.11	1.42	0.181
		26.9	8.52	1.65	0.231
Arias et al. (2013)	WDG	0	8.23	0.83	0.163
		24.5	8.18	0.95	0.142
Ferreira et al. (2020)	WDG	0	10.75	1.8	0.17
		15	11.53	1.9	0.17
		30	11.44	2.01	0.18
		45	11.35	1.91	0.17
Luebbe et al. (2012)	WDG	0	11.7	1.9	0.162
		15	11.5	2	0.174
		30	11.5	1.87	0.163
		45	11.4	1.89	0.167
		60	10.5	1.7	0.162
Salim et al. (2014)	MWDGS	0	11.7	2.09	0.179
		16.7	10.7	1.97	0.186
		33.3	12.3	2.01	0.158
		50	11.1	1.94	0.177
Vasconcelos et al. (2007)	WDG	0	8.45	1.5	0.177
		10.2	7.98	1.4	0.175
Watson et al. (2014)	WDGS	0	10.9	1.66	0.153
		10	11.2	1.85	0.165
		20	11.4	1.87	0.164
		30	11.3	1.96	0.173
		40	11.1	1.94	0.176
		50	10.6	1.78	0.169

Abbreviations: WDG = Wet distillers grains; WDGS = Wet distillers grains with solubles; MWDGS = Modified Wet distillers grains with solubles

REFERENCES

Arias, R.P., Unruh-Snyder, L.J., Scholljegerdes, E.J., Baird, A.N., Johnson, K.D., Buckmaster, D., Lemenager, R.P., Lake, S.L., 2013. Effects of feeding corn modified wet distillers grain plus solubles co-ensiled with chopped whole plant corn on heifer growth performance and diet digestibility in beef cattle. Journal of Animal Science 91, 4366–4373. doi:10.2527/JAS.2012-5777

Arias, R.P., Unruh-Snyder, L.J., Scholljegerdes, E.J., Baird, A.N., Johnson, K.D., Buckmaster, D., Lemenager, R.P., Lake, S.L., 2012. Effects of feeding corn modified wet distillers grain plus solubles co-ensiled with direct-cut forage on feedlot performance, carcass characteristics, and diet digestibility of finishing steers. Journal of Animal Science 90, 3574–3583. doi:10.2527/jas.2011-4502

da Rosa e Silva, P.I.J.L., Vilas Boas e Silva, Y.R., Paulino, P.V.R., de Paula Sousa, D., Possamai, A.J., da Freiria, L.B., Rolim, H.C.L., de Castro Dias Júnior, W., da Silva Cabral, L., 2022. Dried distiller's grains for feedlot Nellore cattle fed non-forage-based diets. Tropical Animal Health and Production 54, 1–9. doi:10.1007/s11250-022-03225-4

Ferreira, M., Niehues, M.B., Tomaz, L.A., Baldassini, W., Ladeira, M., Arrigoni, M., Martins, C.L., Gionbelli, T., Paulino, P., Neto, O.R.M., 2020. Dry matter intake, performance, carcass traits and expression of genes of muscle protein metabolism in cattle fed increasing levels of de-oiled wet distillers grains. Animal Feed Science and Technology 269, 114627. doi:10.1016/j.anifeedsci.2020.114627

Luebbe, M.K., Patterson, J.M., Jenkins, K.H., Buttrey, E.K., Davis, T.C., Clark, B.E., McCollum, I.T., Cole, N.A., MacDonald, J.C., 2012. Wet distillers grains plus solubles concentration in steam-flaked-corn-based diets: Effects on feedlot cattle performance, carcass characteristics, nutrient digestibility, and ruminal fermentation characteristics. Journal of Animal Science 90, 1589–1602. doi:10.2527/jas.2011-4567

Salim, H., Wood, K.M., McEwen, P.L., Vandervoort, G., Miller, S.P., Mandell, I.B., Cant, J.P.,

Swanson, K.C., 2014. Influence of feeding increasing level of dry or modified wet corn distillers grains plus solubles in whole corn grain-based finishing diets on growth performance, carcass traits, and feeding behavior in finishing cattle. Livestock Science 161, 53–59. doi:10.1016/j.livsci.2013.12.020

Schoonmaker, J.P., Claeys, M.C., Lemenager, R.P., 2013. Effect of increasing distillers grains inclusion on performance and carcass characteristics of early-weaned steers. Journal of Animal Science 91, 1784–1790. doi:10.2527/JAS.2011-5075

Uwituze, S., Parsons, G.L., Shelor, M.K., Depenbusch, B.E., Karges, K.K., Gibson, M.L., Reinhardt, C.D., Higgins, J.J., Drouillard, J.S., 2010. Evaluation of dried distillers grains and roughage source in steam-flaked corn finishing diets. Journal of Animal Science 88, 258–274. doi:10.2527/jas.2008-1342

Vasconcelos, J.T., Shaw, L.M., Lemon, K.A., Cole, N.A., Galyean, M.L., 2007. Effects of Graded Levels of Sorghum Wet Distiller's Grains and Degraded Intake Protein Supply on Performance and Carcass Characteristics of Feedlot Cattle Fed Steam-Flaked Corn-Based Diets. Professional Animal Scientist 23, 467–475. doi:10.1532/S1080-7446(15)31007-X

Watson, A.K., Vander Pol, K.J., Huls, T.J., Luebbe, M.K., Erickson, G.E., Klopfenstein, T.J., Greenquist, M.A., 2014. Effect of dietary inclusion of wet or modified distillers grains plus solubles on performance of finishing cattle. Professional Animal Scientist 30, 585–596. doi:10.15232/pas.2013-01302
